# Multidisciplinary treatment of primary intracranial yolk sac tumor: A case report and literature review

**DOI:** 10.1097/MD.0000000000025778

**Published:** 2021-05-14

**Authors:** Zhen-Ning Xu, Xiang-Yong Yue, Xiao-Ci Cao, Ya-Dong Liu, Bao-Shuan Fang, Wen-Hao Zhao, Chen Li, Shuai Xu, Ming Zhang

**Affiliations:** a Department of Radiation Oncology, Hebei General Hospital, Shijiazhuang; b Graduate School of North China University of Science and Technology, Tangshan, China.

**Keywords:** chemotherapy, intensity modulated radiation therapy, multidisciplinary team, stereotactic radiosurgery, surgery, targeted therapy, yolk sac tumor

## Abstract

**Rationale:**

Intracranial yolk sac tumors (YSTs) are rare malignancies with limited treatment options and a dismal prognosis. They are usually managed with surgical resection and chemoradiotherapy.

**Patient concerns:**

Here, we report a patient with primary YST in the pineal region who achieved long term survival. Despite undergoing treatment, he experienced several recurrences over a 15-year period.

**Diagnosis:**

Brain magnetic resonance imaging (MRI) demonstrated the presence of space-occupying lesions in the pineal region and the medial tail of the left lateral ventricle. The tumors were excised, and the histological diagnosis suggested an intracranial YST.

**Interventions:**

The patient achieved long term survival after combined modality therapy including surgery, stereotactic radiosurgery (SRS)/intensity modulated radiation therapy (IMRT), chemotherapy, and targeted therapy.

**Outcomes:**

The disease remained stable. However, the patient gave up treatment and passed away in October 2020, with a total survival of about 15 years.

**Lessons:**

To the best of our knowledge, this patient with intracranial YST had received a longer survival compared with other published reports. We summarize previously published reports of intracranial YST and discuss the importance of multidisciplinary treatment. SRS may have a role, as a focal boost to residual tumor after resection or in case of recurrence after conventional radiotherapy, in the multimodality management of intracranial YSTs.

## 1 Introduction

Yolk sac tumors (YST) is a rare kind of malignant germ cell tumor that mostly occur in children and adolescents.^[1]^ YSTs originate mainly from the gonads, and only about 10% to 20% occur outside the gonads,^[2]^ such as mediastinum,^[3]^ retroperitoneum,^[4]^ central nervous system (CNS),^[5]^ and sacrococcygeal region.^[6]^ Intracranial YST that is commonly localized in the pineal region, saddle and third ventricle, always develop rapidly, and are prone to recurrence and metastasis. It has a poor prognosis, with the 3-year survival of 27.3%.^[7]^ The present study reports the case of a male adult patient with primary YST in the pineal region who survived for up to 15 years after the multimodality treatment approach including surgery, stereotactic radiosurgery (SRS)/intensity modulated radiation therapy (IMRT), chemotherapy, and targeted therapy. To the best of our knowledge, this patient had a longer overall survival after combined modality therapy compared with other published reports.

## 2 Case report

### 2.1 Informed consent

Informed consent for publication of the case details and accompanying images has been obtained from the patient's next of kin.

### 2.2 Case introduction

**Figure 1:**
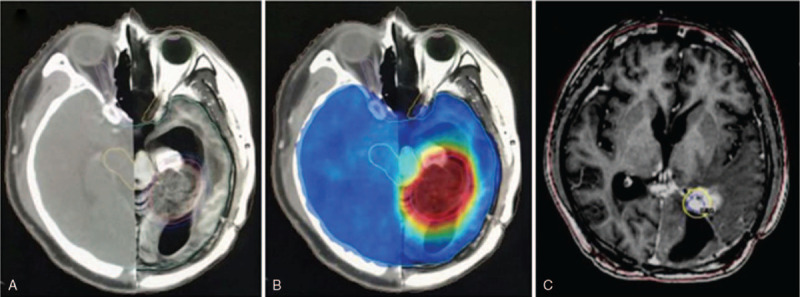
A: The clinical target volume and organs at risk were delineated in the CT-MR fusion images. B: Colorwash of dose distribution within the patient. The 95% of prescribed dose of blue area represents the treatment volume of whole-brain is 36 Gy, and the 95% of prescribed dose of red area represents the treatment volume of tumor is 58 Gy. C: SRS to the left lateral ventricle for the second stage. The yellow line represents the treatment volume, and the blue line represents the 13 Gy isodose line. SRS = stereotactic radiosurgery.

**Figure 2:**
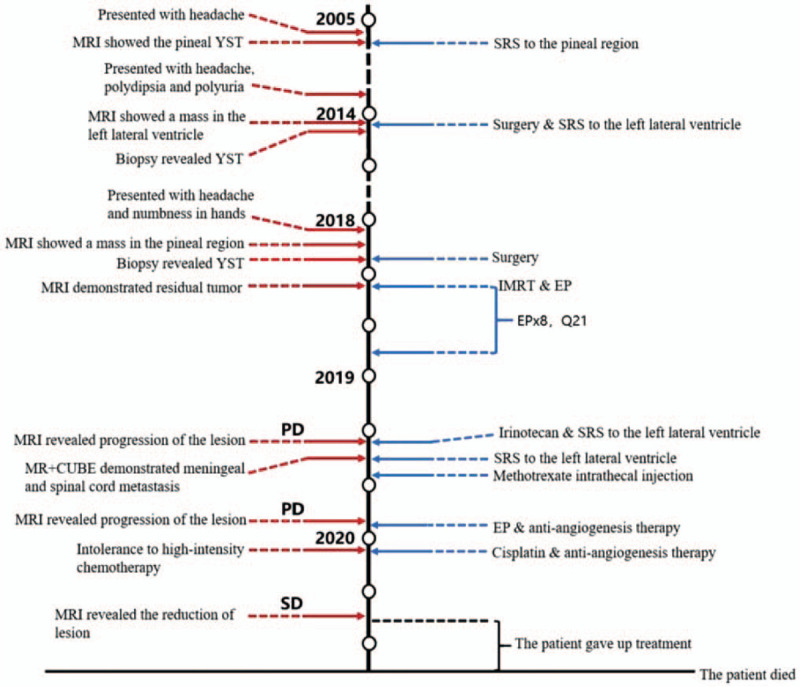
The clinical treatment timeline.

A 20-year-old man was admitted to the hospital because of headache in 2005, and then diagnosed as the pineal YST. Following SRS, the patient's symptom was relieved. In late 2013, he presented with symptoms of headache, polydipsia, and polyuria. Brain magnetic resonance imaging (MRI) demonstrated the presence of a mass in the medial tail of the left lateral ventricle, which was confirmed to be a YST by the postoperative pathology. SRS was subsequently performed after tumor resection. In January 2018, the patient experienced headache again and numbness in hands. MRI was performed and showed space-occupying lesions in the pineal region. The tumor was excised, and the histological diagnosis suggested an intracranial YST, therefore recurrence was considered. One month after the operation, serum alpha-fetoprotein (AFP) was 1230 ng/mL, and MRI showed irregular masses in the left medial temporal lobe, the left parietal lobe, and the trigone and the medial side of left lateral ventricle. Considering the presence of residual lesion and cytological examination of cerebrospinal fluid (CSF) remaining negative, whole-brain radiotherapy (36 Gy/18f) and boost to a total dose of 58 Gy were given (shown in Fig. 1A and B). Meanwhile, systemic chemotherapy was initiated with 8 cycles of EP (etoposide and cisplatin). Regular review during chemotherapy showed that serum AFP continued to decrease to normal and the lesion continued to shrink to stable. MRI led to an evaluation of progression of disease (PD) in April 2019, and 3 cycles of irinotecan with concomitant SRS were administered in subsequent courses. In view of the patient's prior SRS of the left lateral ventricle and the proximity of the lesions to important structures, staged SRS was adopted. The 50% dose curve for the first stage was 13 Gy, and the same dose was given for the second stage 2 months later (shown in Fig. 1C). Because imaging suggested meningeal and spinal cord metastasis, the patient underwent ommaya balloon implantation and methotrexate intrathecal injection. In November 2019, imaging suggested PD recurrence. Due to his intolerance, after 2 cycles of EP combined with anti-angiogenesis, chemotherapy was changed to cisplatin combined with anti-angiogenesis until June 2020, and the patient achieved stable disease (SD). Unfortunately, in the following 4 months the patient gave up treatment and passed away in October 2020, with a total survival of about 15 years. Summary of treatment history is shown in Fig. 2.

## 3 Discussion

The World Health Organization (WHO) divided intracranial germ cell tumors (IGCTs) into 2 fundamental types according to tissue subtypes: germinomas and non-germinomatous germ cell tumors (NGGCTs). NGGCTs include embryonal cancer, yolk sac tumor, choriocarcinoma, and teratoma.^[8]^ Intracranial YSTs account for only 5% to 7% of IGCTs,^[9]^ but generally have very poor survival rates. There are few reports about primary intracranial YSTs. According to the literature of the past 10 years, the age of the patients ranged from 1 to 45 (mean 11) years old (shown in Table 1). Even with multimodality approach to treatment including surgery, radiotherapy and chemotherapy, 3-year survival rate of the YSTs was only 27.3%. In this case, the onset age was 20 years old, and the survival period was as long as 15 years, which is the longest one of intracranial YSTs compared with previously published reports.

**Table 1:** Characteristic of cases of intracranial yolk sac tumor.

Year	Sex	Age, yr	Location	Op	CT	RT	OS, mo
2020^[1]^	M	3	The Fourth ventricle	Yes	No	No	NR
2017^[10]^	M	23	Pineal region	No	Yes	No	NR
2017^[11]^	M	17	Hypothalamic and Pineal region	Yes	Yes	Yes	Alive
2014^[12]^	M	2	Cerebellar vermis	Yes	Yes	No	NR
2014^[13]^	F	45	The anterior third ventricle	Yes	No	Yes	Alive
2014^[14]^	M	3	Left cerebellar hemisphere	Yes	No	No	6
2013^[15]^	F	17	Bilateral basal ganglia	Yes	Yes	Yes	Alive
2013^[16]^	M	4	Posterior fossa	Yes	Yes	Yes	60
2013^[17]^	M	2.5	Posterior fossa	Yes	Yes	Yes	Alive
2012^[18]^	F	15	Pineal region, the right lateral ventricle	Yes	Yes	Yes	NR
2011^[19]^	M	13	Right basal ganglia	No	Yes	Yes	21
2011^[20]^	M	2	Left temporoparietal lobe	Yes	No	No	36
2011^[5]^	M	22	Petrous apex	Yes	Yes	Yes	15

CT = chemotherapy, NR = no report, Op = operation, OS = overall survival, RT = radiotherapy.

Without relevant guidelines currently, the treatment of YST is similar to the treatment of NGGCTs, for which a combination of surgical resection, chemotherapy, and radiotherapy is typically used.^[21]^ Complete surgical resection is the key to treatment, and postoperative chemoradiotherapy is given if tumor is residual.^[22]^ The efficacy of platinum-based chemotherapy (carboplatin or cisplatin) in the treatment of IGCTs has been well established.^[23–25]^ And substituting cisplatin for carboplatin may contribute to a better survival in patients with intracranial NGGCT, which is based on the observation that the latter was less effective than the former at equivalent doses in 2-drug and 3-drug treatment protocols in patients with non-CNS germ cell tumors.^[26]^ At present, conventional 3D conformal radiotherapy/IMRT is mostly used for postoperative radiotherapy, and there are few reports on the treatment of YSTs by SRS.^[27]^

Thanks to the technical characteristic of large dose in targeted area and low dose to the surrounding area, SRS can precisely kill the tumor and reduce the damage to the surrounding normal brain tissues.^[28]^ Therefore, it is increasingly used as primary or adjuvant therapy for pineal tumors.^[29]^ In the case of tumor recurrence in situ and failure to perform conventional radiotherapy, SRS has obvious advantages.^[30]^

The patient received whole-brain radiotherapy and focal radiotherapy and 4 SRS over a 15-year period. Nine years after the first SRS performed in 2005, no tumor recurrence was observed. After recurrence, administering surgery combined with SRS, the local control duration was up to 4 years. The patient received SRS for 3 times in the left lateral ventricle, especially after recurrence in 2019. Considering the patient's tolerance and important adjacent structures, staged SRS was adopted to reduce the radiation damage to the surrounding normal tissues on the basis of ensuring the implementation of an effective and enough biological dose. It can not only achieve local control, but also reduce postoperative adverse events.^[31]^ With the development of radiotherapy equipment and technology, researchers are also exploring new forms of radiation, such as proton radiation.^[32]^

**Figure 3:**
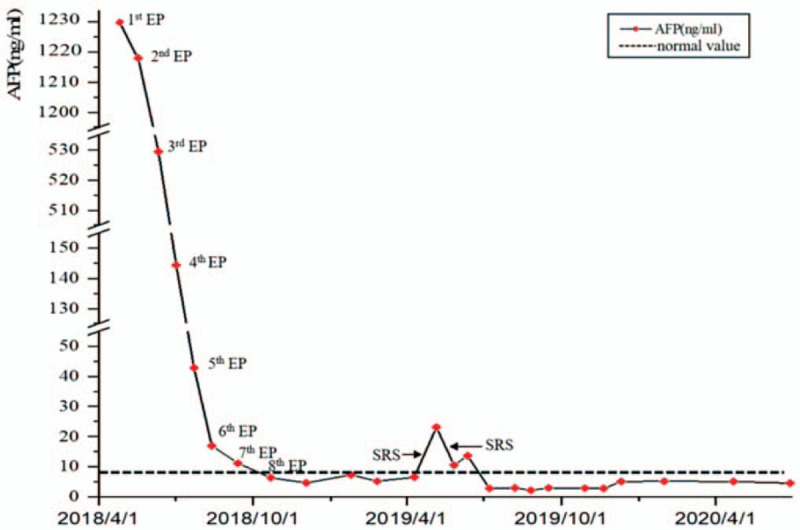
Change in serum AFP levels during the treatment. AFP = alpha-fetoprotein.

Treatment optimization and whole-process management of patients are required. In the process of management, the strategies of efficacy evaluation include tumor markers and imaging. Serum AFP level is a common indicator for evaluating the treatment response to YSTs.^[7]^ Studies have shown that serum AFP >1000 ng/mL at the time of diagnosis is a risk factor for subsequent recurrence, and it is recommended that patients receive intensive chemotherapy.^[33]^ In our case, the serum AFP level dropped constantly to the normal range during the 8 cycles of EP chemotherapy (Fig. 3) and maintained stable because of close monitoring and evaluation through imaging studies, followed by appropriate intervention such as SRS, chemotherapy targeted therapy. At the late stage, management was interrupted due to abandonment of the treatment, and the patient passed away 4 months later, indicating the importance of close monitoring and timely intervention.

## 4 Conclusion

Intracranial YSTs is clinically rare, with high degree of malignancy and poor prognosis. A combination of surgical resection and chemoradiotherapy is recommended for YSTs. However, long term adverse events of conventional radiotherapy are significant. The use of SRS as a focal boost to residual tumor after resection or in case of recurrence after conventional radiotherapy, may improve local control and survival.

With the development of treatment options and drugs, the prognosis of patients has been improved. Evidence-based medicine shows that multidisciplinary treatment (MDT) can improve clinical outcomes. MDT has been considered necessary for patients with cancer. In the course of this patient's management, we chose more effective pattern and drugs through MDT approach, closely monitored as well as timely adjusted therapeutic regimens, which may contribute to improving local control and survival of the patient.

## Author contributions

**Conceptualization:** Ming Zhang.

**Data curation:** Bao-Shuan Fang, Wen-Hao Zhao, Chen Li, Shuai Xu.

**Formal analysis:** Zhen-Ning Xu, Xiang-Yong Yue, Xiao-Ci Cao, Ya-Dong Liu, Ming Zhang.

**Funding acquisition:** Ming Zhang.

**Investigation:** Zhen-Ning Xu, Xiang-Yong Yue, Xiao-Ci Cao.

**Methodology:** Zhen-Ning Xu, Xiang-Yong Yue, Xiao-Ci Cao, Ming Zhang.

**Validation:** Bao-Shuan Fang, Wen-Hao Zhao, Ming Zhang.

**Visualization:** Zhen-Ning Xu, Ya-Dong Liu, Ming Zhang.

**Writing – original draft:** Zhen-Ning Xu, Xiang-Yong Yue, Ming Zhang.

**Writing – review & editing:** Zhen-Ning Xu, Xiang-Yong Yue, Ming Zhang.
